# Dynamics of Socioeconomic Risk Factors for Neglected Tropical Diseases and Malaria in an Armed Conflict

**DOI:** 10.1371/journal.pntd.0000513

**Published:** 2009-09-08

**Authors:** Thomas Fürst, Giovanna Raso, Cinthia A. Acka, Andres B. Tschannen, Eliézer K. N'Goran, Jürg Utzinger

**Affiliations:** 1 Department of Public Health and Epidemiology, Swiss Tropical Institute, Basel, Switzerland; 2 Molecular Parasitology Laboratory, Queensland Institute of Medical Research, Brisbane, Australia; 3 School of Population Health, The University of Queensland, Brisbane, Australia; 4 Centre Suisse de Recherches Scientifiques, Abidjan, Côte d'Ivoire; 5 Département de Sociologie, Université de Cocody-Abidjan, Abidjan, Côte d'Ivoire; 6 UFR Biosciences, Université de Cocody-Abidjan, Abidjan, Côte d'Ivoire; Sabin Vaccine Institute, United States of America

## Abstract

**Background:**

Armed conflict and war are among the leading causes of disability and premature death, and there is a growing share of civilians killed or injured during armed conflicts. A major part of the civilian suffering stems from indirect effects or collateral impact such as changing risk profiles for infectious diseases. We focused on rural communities in the western part of Côte d'Ivoire, where fighting took place during the Ivorian civil war in 2002/2003, and assessed the dynamics of socioeconomic risk factors for neglected tropical diseases (NTDs) and malaria.

**Methodology:**

The same standardized and pre-tested questionnaires were administered to the heads of 182 randomly selected households in 25 villages in the region of Man, western Côte d'Ivoire, shortly before and after the 2002/2003 armed conflict.

**Principal Findings:**

There was no difference in crowding as measured by the number of individuals per sleeping room, but the inadequate sanitation infrastructure prior to the conflict further worsened, and the availability and use of protective measures against mosquito bites and accessibility to health care infrastructure deteriorated. Although the direct causal chain between these findings and the conflict are incomplete, partially explained by the very nature of working in conflict areas, the timing and procedures of the survey, other sources and anecdotal evidence point toward a relationship between an increased risk of suffering from NTDs and malaria and armed conflict.

**Conclusion:**

New research is needed to deepen our understanding of the often diffuse and neglected indirect effects of armed conflict and war, which may be worse than the more obvious, direct effects.

## Author Summary

Armed conflict and war and infectious diseases are globally among the leading causes of human suffering and premature death. Moreover, they are closely interlinked, as an adverse public health situation may spur violent conflict, and violent conflict may favor the spread of infectious diseases. The consequences of this vicious cycle are increasingly borne by civilians, often as a hidden and hence neglected burden. We analyzed household data that were collected before and after an armed conflict in a rural part of western Côte d'Ivoire, and investigated the dynamics of socioeconomic risk factors for neglected tropical diseases (NTDs) and malaria. We identified a worsening of the sanitation infrastructure, decreasing use of protective measures against mosquito bites, and increasing difficulties to reach public health care infrastructure. In contrast, household crowding, the availability of soap, and the accessibility of comparatively simple means of health care provision (e.g., traditional healers and community health workers) seemed to be more stable. Knowledge about such dynamics may help to increase crisis-proofness of critical infrastructure and public health systems, and hence mitigate human suffering due to armed conflict and war.

## Introduction

Infectious diseases and violence have been among the leading causes of premature death and disability throughout history [1]. In 2000, for example, the global burden of armed conflict and war – defined as a more or less organized, large-scale form of violence – was estimated at 26.1 million disability-adjusted life years (DALYs). This burden accounted for 1.9% of the total global burden of disease and injury at the beginning of the new millennium. It is anticipated that armed conflict and war will further rise in the ranking of leading causes of global burden; from position 16 in 1990 to position eight in 2020, with an estimated burden of 41.3 million DALYs or 3.0% of the global burden. An alarming 25.2 million DALYs – or 61% – of this predicted burden is expected to occur in sub-Saharan Africa [2]. It is informative to put these worrying statistics into context: the global burden due to the so-called neglected tropical diseases (NTDs) and malaria, in 2002, were estimated at 56.6 and 46.5 million DALYs, respectively [3],[4]. Taken together, the NTDs and malaria currently account for 6.9% of the total global burden of disease.

Armed conflict and infectious diseases may have a mutually reinforcing impact on each other leading to an even more devastating situation for human health. On the one hand, an adverse public health situation may cause or exacerbate social tensions and contribute to the outbreak of armed conflict [5],[6]. On the other hand, more soldiers died because of infectious diseases than of the enemies' weapons throughout history. Advances in the medical sciences though reduced the risk of infection over the past decades, at least for combatants [1]. The civilian deaths, measured as a proportion of all deaths in armed conflict, have been constantly rising over time [7] and many of these deaths may be due to infectious diseases [7]–[10]. However, the impact of conflict on infectious diseases in the civilian population, even though being significant, is still insufficiently studied [1],[11].

To further our understanding of the complex interactions between armed conflict and risk factors for infectious diseases threatening mainly civilian populations, our study carried out in a rural part of western Côte d'Ivoire investigated the impact of an armed conflict on socioeconomic risk factors for NTDs and malaria. We employed two datasets obtained from interviewing a random sample of households shortly before the outbreak of an armed conflict and again after military hostilities ceased one and a half year later. Previous research in the same area confirmed that socioeconomic and demographic factors had a strong leverage on parasitic disease distribution, even stronger than environmental determinants [12].

## Materials and Methods

### Conceptual framework

The consequences of armed conflict and war on public health can be classified as either direct or indirect. The direct effects consist of immediate physical harm to the human body, mainly injuries and killings.

Indirect effects can occur immediately as well as delayed and affect rather the physical, social, and socioeconomic environment of people than directly the people themselves [1],[7],[11]. Indirect effects include, for example, individual [13],[14] and collective socioeconomic losses [15],[16], destroyed infrastructure and biosphere [9],[17], the disruption of public health systems [8]–[10], social dislocation, migration and displacement [18],[19] or psychological trauma [20],[21]. By changing risk profiles, indirect effects may substantially contribute to increased morbidity and mortality rates during and after an armed conflict [22]. It should be noted, however, that the complexity of all interactions may lead to counter-intuitive outcomes. For example, a marked decline in case fatality among hospitalized children was reported during a war in Guinea-Bissau, which was partly explained by improved treatment as a result of better access to drugs funded and distributed by humanitarian aid and non-governmental organizations [23].

**Figure 1:**
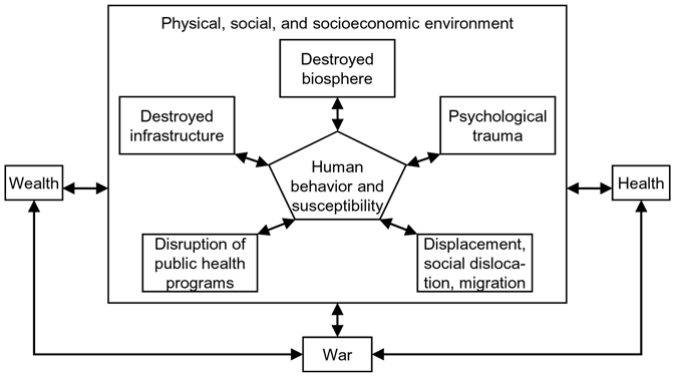
Conceptual framework: direct and indirect effects of an armed conflict on health status of households.

As illustrated in Figure 1, health, wealth and the environment may have a backlash on war by influencing the available resources and conditions for fighting.

### Study area, population and objectives

Our study was carried out between August 2002 and February 2004 in the mountainous region of Man, western Côte d'Ivoire. The area is part of the Ivorian rainforest zone, which belongs to the Western African forest belt. Ecological transformations have occurred in this region over the past decades, spurred by increased demographic pressure due to internal and foreign immigration and the influx of Liberian refugees [24],[25].

In 2002, approximately 250,000 people lived in the study area with about half of them in the regional capital, the city of Man [26],[27]. Most inhabitants belong traditionally to the Yacouba ethnic groups [28], which are involved in a longstanding conflict on land property rights with their southern neighbors, the Krou ethnic groups. Rural households, which are in the main focus of this study, make their living mainly by subsistence farming (rice, cassava, maize, plantain, and yam), the cultivation of a few cash crops (coffee and cocoa), and the keeping of goats, cattle, and poultry. Additionally, there is a small timber industry in the region and some inland fish farming [26],[27].

The area has been subject to intensive fighting during the Ivorian civil war between September 2002 and July 2003. The conflict officially ended by a cease-fire, negotiated with the help of the United Nations (MINUCI/ONUCI mission), but it divided the country in a northern part dominated by the Forces Armées des Forces Nouvelles (FAFN) and a government-held south. After the officially negotiated cease-fire, violence still flared up occasionally. However, at the time this manuscript was drafted (late 2008/early 2009), the borders appeared to be dissolving as demobilization, disarmament, and reintegration efforts were under way even though post-war elections have been postponed several times since the constitutionally scheduled date in 2005.

We focused on the effects of the armed conflict on infrastructure and the health systems, with an attempt to quantify the impact on population density per household, access to clean water and improved sanitation, use of preventive measures, and access to health care. Hence, major risk factors for a number of NTDs, including schistosomiasis, soil-transmitted helminthiases (ascariasis, trichuriasis and hookworm disease), intestinal protozoa infections (entamoebiasis, giardiasis), and malaria were investigated [29]–[32]. NTDs are widespread in our study area and malaria is holoendemic [25]–[27], [33]–[40]. Furthermore, epidemiologic surveys indicated that polyparasitism is the norm rather than the exception in the region of Man [33],[37],[39].

### Data collection

Two cross-sectional surveys were carried out; the first took place in August 2002 and the second in late 2003/early 2004. Thus, the data were collected shortly before the outbreak of the armed conflict and after the officially negotiated cease-fire in an area close to the military front-lines.

Data were obtained using standardized, pre-tested, and locally adapted questionnaires. They were filled out by trained assistants, who interviewed the heads of seven to nine randomly selected households from each of 25 randomly selected villages in the region of Man in the first survey (total 203 households). The seven to nine households interviewed per village equaled approximately 10% of all households in the respective villages. Our study complemented a larger cross-sectional survey done in 55 villages with more than 4,000 schoolchildren screened for *Plasmodium*, *Schistosoma*, and soil-transmitted helminth infections [34]–[37],[40]. In the second survey, the same households were tried to be re-interviewed. Overall, 182 households could be identified again based on (i) location, and (ii) household members.

In both surveys, the heads of households were asked the same questions. The questionnaires started with general demography and some socioeconomic wealth indicators, followed by the main water supply in the village, availability of functioning toilets, and the use of soap. Access to preventive measures was mainly evaluated by asking about the use and availability of protective measures against mosquito bites. For estimating the accessibility of health care, the studied households were asked about the required time and the estimated travel distance to reach a traditional healer, a community health worker, a dispensary, a pharmacy, or a health care centre. An estimated travel time of 5 min was uniformly assigned if the respective household mentioned the existence of the relevant health care infrastructure in the village of residency. Furthermore, if a household reported neither a specific travel time nor the existence of the respective health care infrastructure in the village, the travel time was computed by using the reported distances and information on the generally used means of transportation. In this last case, estimated travel velocities for walking, cycling, and motorized transportation (motorbike, car, or tractor) were 4 km/h, 10 km/h, and 25 km/h, respectively.

### Ethics statement

The study was part of a research project with the objective to contribute to the reconciliation process of Côte d'Ivoire. The project was funded by the Swiss Agency for Development and Cooperation (SDC) and was jointly implemented by the Centre Suisse de Recherches Scientiques (CSRS) and the Université de Cocody-Abidjan (Abidjan, Côte d'Ivoire). The purpose and procedures of the surveys were discussed with local authorities. Oral informed consent was obtained from study participants as the majority of them were illiterate. This was done in the presence of district and regional health and education authorities, with detailed explanations given in the local languages by trained field assistants and local witnesses. This is the usual procedure when administering questionnaires without concurrent collection of biological samples (e.g., blood, stool, and urine) in Côte d'Ivoire and was approved by the institutional research commissions of the Swiss Tropical Institute (STI; Basel, Switzerland) and CSRS. The study was cleared by the national health and education authorities of Côte d'Ivoire. Further details on institutional and organizational background have been presented elsewhere [41].

### Statistical analysis

Data were double entered and cross-checked in EpiInfo version 6.04 (Centers for Disease Control and Prevention; Atlanta, GA, USA). All analyses were done in STATA version 10 (STATA Corporation; College Station, TX, USA). Statistical tests were carried out at 5% significance level. With the exception of the demographic data, all analyses were performed at the household level.

A household asset-based approach was adopted in order to stratify the households into socioeconomic wealth quintiles [42]. Following the instructions of O'Donnell and colleagues [43] (see also revised former technical notes provided by the World Bank's Poverty Reduction and Economic Management (PREM) group and Vyas & Kumaranayake [44]), we used principal component analysis (PCA) and binary information on asset possession and housing characteristics. This approach proved to be valid in rural Côte d'Ivoire and other African settings [45]. The same assets were selected as in our previous work in the same study area [27], [34]–[38].

Poisson and logistic regression models, including random effects accounting for clustering at the village level, and likelihood ratio tests were used in order to check for statistically significant changes and associations.

## Results

### Operational and demographic results

**Figure 2:**
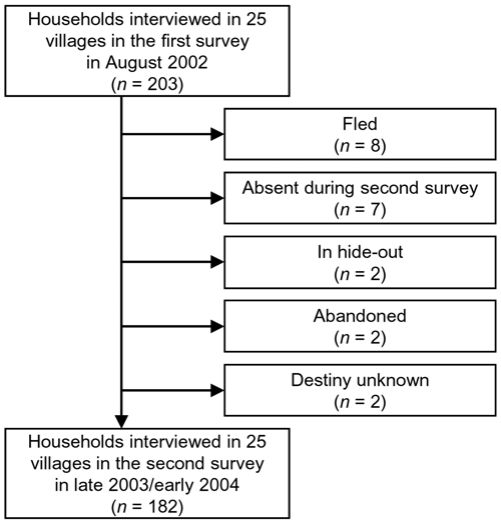
Operational result of the two surveys carried out in August 2002 and late 2003/early 2004 in the region of Man, western Côte d'Ivoire, and the final study sample.

Overall, 203 heads of households in 25 villages were interviewed in the first survey carried out in August 2002. We were able to re-interview 182 household heads one and a half year later. The 21 missing households were explained as follows: inhabitants of eight households fled because of the armed conflict, seven households still existed but no inhabitants were present during our interviews, and two households each were in hide-out near the village, abandoned or disappeared with unknown fate (Figure 2). An attrition analysis, comparing the characteristics of the 21 households that were lost to follow-up with the 182 households that could be re-identified, revealed no differences between these two groups.

Table 1 summarizes demographic findings from the final cohort of 182 households. The number of inhabitants decreased from 1,749 in the first survey to 1,625 in the second. This decrease of 124 individuals (7.1%) was due to migration (*n* = 74) and natural population changes (*n* = 59). Potential misreporting, identified by comparing the reported number of household members in the second survey with the projected number using first survey data and reported information on death, births, and migration, accounted for only nine individuals (0.5% of the original population).

**Table 1:** Population, migration, and natural population changes, stratified by sex and two age groups, in the two surveys carried out in the region of Man, western Côte d'Ivoire.

Demographics		Total number	% of original population	Males (%)	Age<16 years (%)
Total population	First survey	1,749	100	51.4	50.3
	Second survey*	1,625	92.9	51.6	47.4
Population change	Births	36	2.0	55.6	–
	Deaths	−95	−5.4	51.6	25.3
	Immigration	64	3.7	53.1	26.6
	Emigration	−138	−7.9	55.1	39.1

*: Includes a misreporting of nine persons, which equals 0.5% of the original population.

Overall, our data revealed a more mobile male population, but at the end the gender distribution remained virtually unchanged (difference (diff.): 0.2%; 95% confidence interval (CI): −3.1%, 3.5%). Also, the age structure of the studied population did not vary significantly between the two surveys (diff. = −2.9%; CI: −6.2%, 0.4%). However, young people were more likely to have left the study area rather than to in-migrate. In general, an increased rural-urban migration is supported by the fact that 66% of all people who left their villages were supposed to have migrated to an urban setting nearby or far away.

**Figure 3:**
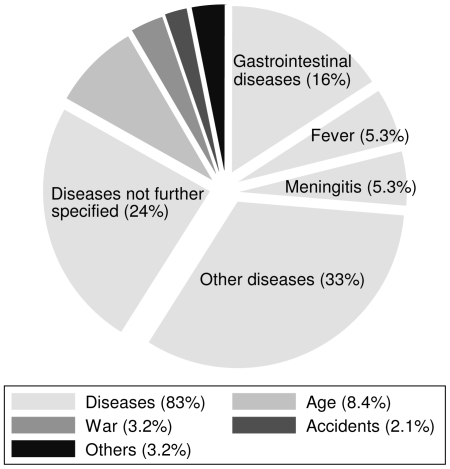
Pie chart illustrating the reported causes of death (in % of all deaths).

Adjusting the natural population change to a calendar year and assuming no temporal variation, we estimated a birth rate of 13.0 per 1,000 persons per annum. The respective death rate was 34.3 per 1,000 persons per annum. Hence, our data imply a birth rate, which was over three times lower, and a death rate, which was almost three times higher than the latest pre-conflict birth and death rates reported for Côte d'Ivoire by two different sources (United Nations Statistics Division [46] and Institut Nationale de la Statistique (INS) of Côte d'Ivoire [47]). These differences are striking, even after taking into consideration the limitation of our own data, and the fact that the latest United Nations' and INS' statistics from 2000 are neither stratified into rural and urban settings nor specific for our study area. However, as shown in Figure 3, only 3 deaths (3.2%) were directly attributed to the armed conflict by the interviewed heads of household. Conversely, diseases were the most frequent causes of death (83%) with gastrointestinal infections (16%), fever (5.3%), and meningitis (5.3%) being the most frequently reported illnesses. More than a quarter of all disease-related deaths lacked further specification.

When asked about the three most important problems since the fighting started, 48% of all households mentioned health-related issues, followed by the lack of food (29%) and the interruption of public services (13%). Most of the households considered the situation of malaria (78%), helmintic diseases in general (72%), and schistosomiasis in particular (51%), as worse than before the armed conflict.

### Household assets ownership and wealth indices

Asset possession followed an intuitively logical trend in both surveys. Possession of ‘goods’ (high-quality housing, electronic devices) increased with increasing wealth and possession of ‘bads’ (low-quality housing) decreased. The absence of land and/or house ownership is often explained as the equivalent of employed work (e.g., teaching) [48] and thus, as it was also the case in our study, plausibly associated with higher socioeconomic status. Interestingly, overall wealth as measured by the sum of assets of all households showed only little variation between the two surveys.

### Crowding

**Figure 4:**
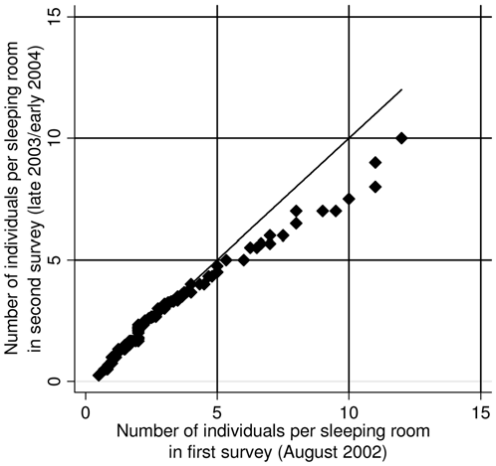
Quantile-quantile plot of the number of individuals per sleeping room in the two surveys carried out in August 2002 and late 2003/early 2004 in the region of Man, western Côte d'Ivoire.

The number of individuals per sleeping room was used as a proxy for crowding. Figure 4 shows the quantile-quantile plot of the number of individuals per sleeping room for the two cross-sectional surveys. Only small changes occurred in population densities in the most crowded households between surveys. The overall decrease in population by 7.1% was almost offset by a decrease of 6.8% of the total number of available sleeping rooms. Using Poisson regression models revealed no statistically significant difference between the population density in the first and in the second survey (*p* = 0.989), but significantly decreasing numbers of individuals per sleeping room with increasing wealth was observed in both surveys (*p*<0.001 for each survey). However, there was no association with the educational level of the head of household (first survey: *p* = 0.570; second survey: *p* = 0.652).

### Water supply and sanitation

Table 2 illustrates the use of different sources of water supply in the villages. The water supply was primarily based on traditional wells in both surveys. Theses traditional wells are typically constructed like draw wells, and hence consist of a simple dug hole in the ground and a bucket on a rope to pull up the groundwater. Other important ways of communal water supply include the collection of rain water, stagnant surface water bodies such as stagnant arms of rivers, multipurpose small dams, and ponds. There are also fountains, which are normally hand-operated groundwater pumps. Less common sources of water supply were use of surface water, natural springs, and tap water. The more sophisticated techniques of fountains and tap water, as well as the use of flowing surface water were significantly less often mentioned in the second survey.

**Table 2:** Households mentioning different sources of water supply in their village of residence in the two surveys carried out in the region of Man, western Côte d'Ivoire (in % of households).

Source of water supply	First survey (August 2002)	Second survey (late 2003/early 2004)
Traditional well	88	88
Rain water	78	69
Stagnant surface water^1^	75	69
Fountains	65	48*
Flowing surface water^2^	62	32*
Natural spring	47	56
Tap water	33	0*

1 Including stagnant arms of rivers, water supply dams, and ponds.

2 Including rivers and creeks.

*: Statistically significant difference between the two surveys, using logistic regression models (*p*<0.05).

Virtually all households used soap (98% in the first and 96% in the second survey). Neither the armed conflict nor the socioeconomic turbulences influenced the availability of soap.

The analysis of the availability of functioning toilets was significantly associated with household wealth in both surveys, as summarized in Table 3. However, in the wake of the armed conflict, the overall proportion of households with cemented toilets significantly decreased and the overall proportion of households with no toilet significantly increased. Two-third of all households reported having only an uncemented latrine or no toilet at all in the second survey. In both surveys, there was no statistically significant association between the availability of different types of toilets and the heads of households' educational level (all *p*>0.05).

**Table 3:** Households reporting different types of toilets in the two surveys carried out in the region of Man, western Côte d'Ivoire (in % of households, stratified by wealth quintiles).

Survey	Wealth quintile	Type of toilet		
		None	Uncemented	Cemented
First survey (August 2002)	Most poor	68*	16	16*
	Very poor	43*	16	41*
	Poor	42*	8.3	50*
	Less poor	25*	8.3	67*
	Least poor	19*	5.6	75*
	Total	40**	11	50**
Second survey (late 2003/early 2004)	Most poor	73*	11	16*
	Very poor	72*	17	11*
	Poor	51*	11	38*
	Less poor	42*	19	39*
	Least poor	22*	11	67*
	Total	52**	14	34**

*: Statistically significant difference between wealth quintiles in the respective survey, using logistic regression models (*p*<0.05).

**: Statistically significant difference between the totals of the two surveys, using logistic regression models (*p*<0.05).

### Use and availability of protective measures against mosquito bites

The main protective measures used by the studied households to avoid mosquito bites included indoor insecticide repellents, mosquito nets (either treated with an insecticide or left untreated), fumigating coils, and natural repellents (plant leaves). The results also revealed that some households used more than just one protective measure and others, which took no action at all.

Table 4 shows that fumigating coils and plant leaves were the most common measures, while insecticide-treated mosquito net coverage was very low in the region in both surveys (<5%). In total, the application of insecticides and fumigating coils was significantly lower in the second survey, whereas the percentage of households mentioning no protection at all increased significantly to an overall level of 39%. The total number of households using multiple protective measures decreased significantly from 25% to 10% (*p*<0.001). Meanwhile, the total percentage of households using mosquito nets, either insecticide-treated or not, and plant leaves did not change significantly.

**Table 4:** Use of different types of protective measures against mosquito bites in the two surveys carried out in the region of Man, western Côte d'Ivoire (in % of households, stratified by wealth quintiles).

Survey	Wealth quintile	Protective measures					
		Insecticide spray	Mosquito net	Insecticide-treated net	Fumigating coil	Plant leaves	None
First survey (August 2002)	Most poor	11	19	0	27	16	43
	Very poor	14	22	5.4	35	27	30
	Poor	17	17	0	53	36	14
	Less poor	28	17	5.6	44	33	19
	Least poor	31	19	8.3	47	11	17
	Total	20**	19	3.9	41**	25	25**
Second survey (late 2003/early 2004)	Most poor	2.7	2.7	0	11	24	62*
	Very poor	11	14	0	31	28	31*
	Poor	8.1	5.4	0	27	30	41*
	Less poor	5.6	19	2.8	31	17	31*
	Least poor	11	22	8.3	25	22	28*
	Total	7.7**	13	2.2	25**	24	39**

*: Statistically significant difference between wealth quintiles in the respective survey, using logistic regression models (*p*<0.05).

**: Statistically significant difference between the totals of the two surveys, using logistic regression models (*p*<0.05).

Socioeconomic position was only associated with using no protection in the second survey. Educational level of the respective heads of households had no influence (all *p*>0.05).

Before the outbreak of the armed conflict, two-third of all heads of households knew a place in their village of residency where they could purchase an insecticide repellent, and a third knew a place where to buy a mosquito net (Table 5). At least one head of household reported a point of sale of insecticide repellent in all 25 villages included in the study and in all but three villages a point of sale of mosquito nets. However, knowing such an opportunity was not associated with wealth in the first survey.

**Table 5:** Knowledge of an opportunity to buy an insecticide spray or a mosquito net in the village of residence in the two surveys carried out in the region of Man, western Côte d'Ivoire (in % of households, stratified by wealth quintiles).

Survey	Wealth quintile	Opportunity to buy	
		Insecticide spray	Mosquito net
First survey (August 2002)	Most poor	54	27
	Very poor	60	27
	Poor	56	14
	Less poor	72	53
	Least poor	86	42
	Total	65*	32*
Second survey (late 2003/early 2004)	Most poor	38	2.7
	Very poor	56	8.3
	Poor	46	5.4
	Less poor	61	5.6
	Least poor	67	8.3
	Total	53*	6.0*

*: Statistically significant difference between the totals of the two surveys, using logistic regression models (*p*<0.05).

After the outbreak of the armed conflict, the known opportunities for preventive measures against mosquito bites decreased dramatically. In the second survey, only half of the interviewed heads of households mentioned an opportunity to purchase an insecticide repellent in their village and only every fifteenth an opportunity to buy a mosquito net. The availability of insecticide spray was confirmed in 23 villages, but the availability of mosquito nets in only six villages. Socioeconomic wealth was not significantly associated with the knowledge of existing selling points in the second survey.

Educational level of the heads of households did not show any significant association with knowing an opportunity to buy an insecticide repellent (first survey: *p* = 0.738; second survey: *p* = 0.780), as well as with knowing an opportunity to buy a mosquito net (first survey: *p* = 0.257; second survey: *p* = 0.404).

### Accessibility of different health care structures

Overall, 19% of all households mentioned insufficient medical care as one of the most severe problems since the beginning of the armed conflict. Table 6 summarizes average travel times to different types of health care structures, stratified by socioeconomic status, in order to better understand accessibility. An arbitrary cut-off point of one hour was used for categorizing and further analyzing the outcomes.

**Table 6:** Travel times from home to different types of health care structures in the two surveys carried out in the region of Man, western Côte d'Ivoire (in % of households, stratified by wealth quintiles).

Survey	Wealth quintile	Traditional healer (%)			Community health worker (%)			Dispensary (%)			Pharmacy (%)			Health care center (%)		
		≤60 min	>60 min	No	≤60 min	>60 min	No answer	≤60 min	>60 min	No answer	≤60 min	>60 min	No answer	≤60 min	>60 min	No answer
First survey (August 2002)	Most poor	65	2.7	32	68	0	32	78	19	2.7	32	35	32	32	8.1	60
	Very poor	73	11	16	76	8.1	16	60	30	11	41	46	14	41	24	35
	Poor	81	2.8	17	86	2.8	11	64	28	8.3	33	36	31	36	31	33
	Less poor	78	0	22	83	2.8	14	83	8.3	8.3	42	28	31	53	14	33
	Least poor	64	5.6	31	64	11	25	78	17	5.6	39	36	25	39	19	42
	Total	72	4.4	24	75	5	20	73**	20	7.1	37**	36**	26	40	19**	41**
Second survey (late 2003/early 2004)	Most poor	65	2.7	32	60	5.4	35	54	30	16	14*	57	30	19	14	68
	Very poor	67	8.3	25	86	5.6	8.3	44	47	8.3	17*	58	25	19	8.3	72
	Poor	68	5.4	27	70	8.1	22	65	24	11	19*	62	19	38	8.1	54
	Less poor	69	5.6	25	83	2.8	14	75	22	2.8	36*	53	11	50	5.6	44
	Least poor	69	14	17	78	0	22	86	11	2.8	53*	36	11	42	2.8	56
	Total	68	7.1	25	75	4.4	20	65**	27	8.2	28**	53**	19	34	7.7**	59**

*: Statistically significant difference between wealth quintiles in the respective survey, using logistic regression models (*p*<0.05).

**: Statistically significant difference between the totals of the two surveys, using logistic regression models (*p*<0.05).

Traditional healers and community health workers showed comparatively good accessibility in both surveys regardless of a household's socioeconomic status, as most households reached them within one hour. Furthermore, the presence of a traditional healer and a community health worker was confirmed by at least one household in all except one village in both surveys. However, about a fifth of all households gave no answer; a fact that may indicate either that these households did not know where the next healer or community health worker was at that time or that they were not able to guess.

Most households reported to reach a dispensary within one hour. However, this proportion decreased significantly between the two surveys. Socioeconomic status seemed to play no role. Longer travel times were noted with respect to pharmacies and their accessibility became worse in the second survey. The proportion of households requiring no more than one hour to reach the nearest pharmacy significantly decreased to an overall level of 28% and the proportion requiring longer significantly increased to 53%. The influence of socioeconomic status became more important in the second survey.

Finally, on average, less than every second household reached a health care centre within one hour in the first survey (40%). This proportion was still twice as high as the proportion of households which needed more than one hour (19%), but about 40% of all households were not able to guess or did not know where the next health care centre was. Both the proportions of households which reported less or more than one hour decreased in the second survey with the latter decrease showing statistical significance. Consequentially, the proportion of households giving no answer increased significantly to 59%. There was no statistically significant association with socioeconomic status.

The educational level of the heads of households was not significantly associated with accessibility in both surveys and for all types of health care structures.

## Discussion

We used two micro-level datasets, originating from cross-sectional household surveys carried out before and after an armed conflict in Côte d'Ivoire, to determine the dynamics of socioeconomic risk factors for NTDs and malaria in the face of military hostilities. Reliable data for such analyses are often lacking due to missing baseline data, security issues, and military sensitivities [49]. Our data are consistent, as could be shown by a high recapture rate of households (90%) and accurate population data (misreporting of only 0.5% of the original population in the second survey).

Several particularities of the present study warrant further discussion. First, a remarkable decrease in the number of available sleeping rooms offset the decrease in the number of household members, and hence the number of individuals per sleeping room remained unchanged. Adaptation of housings could be due to a rearrangement of available rooms as well as war-related collateral damage.

Second, the decrease of safe water provision by tap or fountain [50] increased the households' dependency on traditional wells, stagnant surface water, rain water, and natural springs. Interestingly, the use of flowing surface water decreased, which is likely to result in a reduced risk of schistosomiasis transmission. However, especially traditional wells and stagnant surface water may be at a particular risk of local contamination. In combination with the deteriorating toilet standards, water-related NTDs such as intestinal protozoa infections may further spread. In addition, the anticipated increase in open defecation leads to increased soil contamination, and hence to conditions favorable for the transmission of soil-transmitted helminths [29].

Third, while the use of the most expensive (i.e., insecticide-treated and untreated mosquito nets) and the least expensive (i.e., burning of plant leaves) protective measure against mosquito bites did not vary significantly between the two surveys, the reduced use of insecticides, fumigating coils, and multiple protection as well as the increased proportion of households using no protection at all suggests an increased risk of being bitten by mosquitoes and consequently an increased risk for malaria transmission. Furthermore the availability of insecticide repellents or mosquito nets in the respective villages seemed to have become restricted. A shortfall on the supply side of malaria protection may be an instructive example also with regard to other protective and health improving measures.

Fourth, the evaluation of accessibility of the different types of health care structures revealed that the travel times to reach traditional healers and community health workers were comparatively short, equitably distributed over wealth quintiles, and stable over the two surveys, which may also indicate robust resilience to crisis. Thus, one might consider the integration of traditional healers and community health workers into the health systems.

Previous studies confirmed a worsened accessibility, especially of more sophisticated health care infrastructures [51] and emphasized the importance of more equity-balanced health care, which could serve as a strategy for poverty alleviation in the region of Man and elsewhere in sub-Saharan Africa [35].

There are several aspects of our study that need to be discussed in further depth. Most important, the study was only looking at one dimension, namely accessibility, of the broader concept of access to health care as described by Penchansky & Thomas [52]. Furthermore, it did not take the quality of the respective health care infrastructure or the health provision efforts of “exogenous” organizations such as the International Committee of the Red Cross (ICRC) or Médecins Sans Frontières (MSF) into consideration. In fact, the presence of organizations providing emergency relief can have major positive effects on human health in armed conflict settings [23].

Additionally, while measuring accessibility in terms of reported travel times has some advantages, it also has important drawbacks. On the one hand, reported travel times may better reflect effective distances between a household and a health care delivery centre than shortest straight-line distances derived from remote sensing and geographic information systems (GIS). This should be especially true in difficult-to-access areas like our mountainous study area. However, previous remote sensing and GIS analysis already showed significant association between shortest straight-line distances to the nearest facility and household wealth in the study area, demonstrating the validity even of this rather simple approach [35].

On the other hand, the used approach resulted in a high proportion of households giving no answer. This outcome is difficult to interpret and was previously explained by households, which are uninformed about the location of the relevant health care structures or which are simply not able to reasonably guess. However, giving no answer was not significantly associated with education and this result may support the hypothesis that rather the missing information about the location than the inability to guess was the problem. If this is the case, the high proportion of households giving no answer could be interpreted as a lack of knowledge and consequently a risk factor for ill-health.

The average educational level of the household heads, often reported as an additional risk factor [29], did not significantly change over time and also showed no association with the other risk factors under investigation. This may be explained by a low educational attainment with approximately 60% of the heads of households reporting to have never attended school.

Wealth, in contrast, had a positive effect, which means that richer households could constrain at least some of the risk factors (e.g., less crowded living conditions, enhanced availability of functioning toilets, higher proportion of people using protective measures against mosquito bites, and shorter travel times to pharmacies). Pre-conflict studies conducted in the same study area also revealed a somewhat lower level of hookworm infection among richer households [35],[36]. However, households of all wealth quintiles seem to be negatively struck by the armed conflict, and hence confronted with aggravated risk profiles.

A survey conducted in the same area by the Ministry of Health, UNICEF, and WHO described similar patterns of water supply and toilet standards as the present study, but found no changes in these variables over time. In the aforementioned report, the precarious sanitarian conditions were emphasized, along with lower mortality rates and a lower proportion of disease-related deaths in the face of the armed conflict [53]. Possible explanations for the somewhat differing findings include study design issues and differences in the exact classification of water supply and sanitation facilities. The present study used two consecutive cross-sectional surveys of the same rural households, whereas the other study was based on one cross-sectional survey with retrospective questions, which leads to a higher risk of recall bias. Also, data about mortality and causes of death included figures from rural as well as urban areas. Nevertheless, the derived recommendations from the two studies are congruent and include the securing of the livelihoods of the local population, the improvement of the water supply, toilet standards, use and availability of preventive measures, as well as the rehabilitation of the health systems on all administrative levels by taking into account the experiences made during the armed conflict.

It follows that the risk for NTDs and malaria has considerably increased in the rural part of the region of Man. Given that the surveys are separated by a violent conflict, and the second survey carried out in an environment of socioeconomic turmoil, it seems likely that the occurrence of violent conflict, and not some other cause, is at the origin of this observation. Although the study did not investigate the actual direct causes of a riskier situation for the population of Man, e.g., why tap water stopped flowing or why overflowing cemented latrines were replaced by simple earthen ones, it is conceivable that the conflict situation caused an excess risk for NTDs and malaria. First, the conflict has been the most dominant aspect of daily life in western Côte d'Ivoire between the two surveys. Second, the pace at which the changes have occurred points clearly to a severe disruption of the socioeconomic tissue, which has been caused by the conflict. Third, our findings are consistent with the perceptions of the household heads about increased health-related problems in the households, the spread of schistosomiasis, other helminthic diseases and malaria, the interruption of public health services and the given information about increased socioeconomic risk factors since the fighting started. Hence, the excess cases of death among the rural civilians might be attributable to indirect effects of the armed conflict and the indirect character of this association may explain the very low fraction of deaths attributed to war by the interviewed heads of households. However, to unambiguously prove the causality of the above mentioned facts is very difficult, even though a plausible causal chain emerged. During the troubled times of an armed conflict, it is impossible to account for all potential confounders, especially if one thinks about the many theoretical and practical challenges that researchers face under such highly dynamic and unpredictable circumstances.

An important lesson from our case study is the need for additional micro-level research investigating not only direct but also indirect effects of armed conflict and war, as indirect effects may be at least as important as the direct ones and often affect particularly the civilian population. A growing body of literature demonstrates the feasibility of war-related research [54]–[56]. Additional information may help to design critical infrastructure and public health systems that are more crisis-proof. The potential gains of crisis-proof public health systems in terms of reduced human suffering are immense and should provide enough motivation for future scientific efforts.

## Supporting Information

Alternative Language Abstract S1 French translation of the abstract by ABT.
(0.02 MB DOC)

Alternative Language Abstract S2 German translation of the abstract by TF.
(0.03 MB DOC)

Alternative Language Abstract S3 Italian translation of the abstract by GR.
(0.02 MB DOC)
