# Supplementary material for: Dynamics of Socioeconomic Risk Factors for Neglected Tropical Diseases and Malaria in an Armed Conflict
Source: PLoS Negl Trop Dis. 2009 Sep 8;3(9):e513. doi: 10.1371/journal.pntd.0000513 (PMC2731884; doi:10.1371/journal.pntd.0000513)
Supplement: Alternative Language Abstract S1 — French translation of the abstract by ABT. (0.02 MB DOC) [file pntd.0000513.s001.doc]

**Maladies Tropicales Négligées et Malaria dans les conflits armés: dynamique des facteurs de risques socio-économiques**

**Résumé**

***Introduction :*** Les conflits armés et la guerre font partie des causes principales d’infirmité et de mort prématurée et les civils représentent une part croissante des personnes touchées. Les dommages infligés aux civils relèvent principalement d’effets indirects ou d’impacts collatéraux, au nombre desquels l’augmentation du risque de contracter une maladie infectieuse. Nous avons étudié la dynamique des facteurs de risques socioéconomiques des maladies tropicales négligées et du paludisme dans des communautés rurales de la région de Man à l’Ouest de la Côte d’Ivoire, qui était une scène des hostilités sévères pendant la guerre civile de 2002/2003.

***Méthodologie :*** Des questionnaires identiques, standardisés et précédemment testés ont été soumis aux chefs de 182 ménages dans 25 villages peu avant et juste après le conflit armé de 2002/2003.

***Résultats Principaux :*** Le surpeuplement des ménages, calculé sur la base du nombre de personnes par chambre à coucher, n’a pas été modifié suite à la période de conflit. Cependant, les installations sanitaires, déjà insatisfaisantes au départ, la disponibilité et l’utilisation des mesures préventives contre les piqûres de moustiques ainsi que l'accessibilité aux points de soins de santé se sont tous vus détériorés. L’explication directe de ces observations par le conflit reste incomplète, ceci notamment dû à l’environnement de recherche peu propice. Bien que cette chaîne causale soit incomplète, la chronologie de l’étude, d’autres sources et des évidences anecdotiques laissent supposer une relation entre le conflit armé et un plus grand risque de souffrir d’une maladie tropicale négligée ou du paludisme.

***Conclusion :*** De nouvelles recherches sont nécessaires pour approfondir notre compréhension des effets indirects, diffus et négligés des conflits armés et de la guerre, qui peuvent être pires que les effets plus évidents et directs.

***Traduction:*** Andres B. Tschannen
