# Supplementary material for: Dynamics of Socioeconomic Risk Factors for Neglected Tropical Diseases and Malaria in an Armed Conflict
Source: PLoS Negl Trop Dis. 2009 Sep 8;3(9):e513. doi: 10.1371/journal.pntd.0000513 (PMC2731884; doi:10.1371/journal.pntd.0000513)
Supplement: Alternative Language Abstract S2 — German translation of the abstract by TF. (0.03 MB DOC) [file pntd.0000513.s002.doc]

**Dynamik von sozioökonomischen Risikofaktoren für vernachlässigte Tropenkrankheiten und Malaria in einem bewaffneten Konflikt**

**Zusammenfassung**

***Hintergrund:*** Bewaffnete Konflikte und Kriege sind wichtige Ursachen für Invalidität und frühzeitigen Tod und der Anteil verletzter oder getöteter Zivilisten in Konfliktsituationen nimmt laufend zu. Der Hauptschaden für Zivilisten hat indirekte Ursachen, wie zum Beispiel veränderte Risikoprofile für Infektionskrankheiten. Wir konzentrierten uns auf ländliche Dorfgemeinschaften im Westen der Elfenbeinküste, wo während dem ivorianischen Bürgerkrieg (2002/2003) heftig gekämpft wurde, und untersuchten die Dynamik von sozioökonomischen Risikofaktoren für vernachlässigte Tropenkrankheiten und Malaria.

***Methoden:*** Identische, standardisierte und im Voraus getestete Fragebogen wurden kurz vor und nach dem bewaffneten Konflikt von 2002/2003 mit den Haushaltsvorständen von 182 zufällig ausgewählten Haushalten in 25 Dörfern in der Region von Man im Westen der Elfenbeinküste ausgefüllt.

***Wichtigste Ergebnisse:*** Es konnte kein Unterschied in der Wohnungsbelegung, d.h. der Anzahl Personen pro Schlafraum, festgestellt werden, aber die bereits vor dem Konflikt ungenügende sanitäre Infrastruktur, die Verfügbarkeit und Anwendung von Präventivmassnahmen gegen Moskitostiche, sowie die Erreichbarkeit von medizinischen Versorgungseinrichtungen verschlechterten sich. Die Kausalität zwischen unseren Erkenntnissen und dem Konflikt sind unvollständig, was teilweise auch durch die schwierigen Arbeitsbedingungen im Konfliktgebiet erklärt werden kann. Der zeitliche Ablauf, die Verhältnisse bei den Befragungen und zusätzliche Informationsquellen und Einzelberichte weisen jedoch auf einen Zusammenhang zwischen einem grösseren Risiko an vernachlässigten Tropenkrankheiten und Malaria zu leiden und dem bewaffneten Konflikt hin.

***Schlussfolgerung/Bedeutung:*** Weitere Forschungsanstrengungen sind notwendig um unser Verständnis der häufig diffusen und vernachlässigten, indirekten Auswirkungen von bewaffneten Konflikten und Kriegen, welche schlimmer sein können als die offensichtlicheren, direkten Effekte, weiter zu vertiefen.

***Übersetzung:*** Thomas Fürst
