# Supplementary material for: Dynamics of Socioeconomic Risk Factors for Neglected Tropical Diseases and Malaria in an Armed Conflict
Source: PLoS Negl Trop Dis. 2009 Sep 8;3(9):e513. doi: 10.1371/journal.pntd.0000513 (PMC2731884; doi:10.1371/journal.pntd.0000513)
Supplement: Alternative Language Abstract S3 — Italian translation of the abstract by GR. (0.02 MB DOC) [file pntd.0000513.s003.doc]

**Le dinamiche dei fattori di rischio socioeconomici associati alle malattie tropicali trascurate ed alla malaria**

**Riassunto**

***Introduzione:*** Conflitto armato e guerra sono cause principali di disabilità e morte precoce. I civili uccisi o feriti durante conflitti armati sono in aumento. Un maggior numero di civili soffrono degli effetti indiretti o impatti collaterali, come ad esempio il cambiamento dei rischi per contrarre malattie infettive. Il nostro lavoro focalizza sulle comunità rurali nella regione del Man, nel ovest della Costa d’Avorio, dove una guerra civile ebbe luogo nel 2002/2003, per valutare le dinamiche dei fattori di rischio socioeconomici associati alle malattie tropicali trascurate ed alla malaria.

***Metodologia:*** Dei questionari standardizzati e pre-testati sono stati amministrati a 182 capi famiglia selezionati a caso in 25 villaggi nella regione del Man prima e dopo il conflitto armato che ebbe luogo nel 2002/2003.

***Risultati principali:*** Non è stata trovata nessuna differenza di affollamento, misurato attraverso il numero di occupanti per stanza, ma è stato trovato un peggioramento dei servizi igienico-sanitari dopo il conflitto. Inoltre è stato trovato un deterioramento nell’uso delle misure protettive contro le punture di zanzara e l’accessibilità alle strutture sanitarie. Sebbene la catena causale tra questi risultati ed il conflitto è incompleta, parzialmente sbiegabile dalle difficoltà incontrate durante il lavoro in zona di conflitto, il tempismo dell’ indagine e l’evidenza aneddotica puntano verso una relazione tra un aumento del rischio per contrarre malattie tropicali trascurate e la malaria ed un conflitto armato.

***Conclusione:*** Nuova ricerca è necessaria per approfondire la comprensione degli effetti indiretti e spesso trascurati, causati da conflitti armati e guerre, che potrebbero essere peggiori degli effetti diretti immediati.

***Traduzione:*** Giovanna Raso
